# Antibiotic susceptibility profiles of *Mycoplasma bovis* strains isolated from cattle in Hungary, Central Europe

**DOI:** 10.1186/s12917-014-0256-x

**Published:** 2014-10-25

**Authors:** Kinga M Sulyok, Zsuzsa Kreizinger, Lilla Fekete, Veronika Hrivnák, Tibor Magyar, Szilárd Jánosi, Nóra Schweitzer, Ibolya Turcsányi, László Makrai, Károly Erdélyi, Miklós Gyuranecz

**Affiliations:** Institute for Veterinary Medical Research, Centre for Agricultural Research, Hungarian Academy of Sciences, Hungária körút 21, Budapest, 1143 Hungary; Veterinary Diagnostic Directorate, National Food Chain Safety Office, Tábornok utca 2, Budapest, 1143 Hungary; Faculty of Veterinary Science, Szent István University, Hungária körút 23-25, Budapest, 1143 Hungary

**Keywords:** Antibiotic resistance, MIC, Fluoroquinolones, Microbroth dilution, *Mycoplasma bovis*

## Abstract

**Background:**

*Mycoplasma bovis* is a worldwide pathogen, causative agent of pneumonia, mastitis, arthritis, and a variety of other symptoms in cattle. The economic losses due to mycoplasma pneumonia could be reduced by antibiotic treatment. The aim of the present study was to determine the *in vitro* susceptibility of *M. bovis* strains isolated from cattle in Hungary to eleven antibiotics.

**Results:**

Minimal inhibitory concentration (MIC) values of 35 *M. bovis* strains collected from different parts of Hungary between 2010 and 2013 were determined by the microbroth dilution method. Strains with high MIC values were found in the case of all applied antibiotics. The most effective antibiotics tested *in vitro* were fluoroquinolones (MIC_90_ danofloxacin 0.312 μg/ml, enrofloxacin 0.312 μg/ml, marbofloxacin 0.625 μg/ml). Our results confirm the observations of increasing MIC values to antibiotics commonly used in the therapy of mycoplasma infections, primarily to tetracyclines; tetracycline (MIC_90_ 16 μg/ml) and oxytetracycline (MIC_90_ ≥ 64 μg/ml) and macrolides; tylosin (MIC_90_ ≥ 128 μg/ml) and tilmicosin (MIC_90_ ≥ 128 μg/ml). The growth of many *M. bovis* strains was not inhibited by gentamicin (MIC_90_ 8 μg/ml), spectinomycin (MIC_90_ ≥ 256 μg/ml), florfenicol (MIC_90_ 8 μg/ml) or lincomycin (MIC_90_ ≥ 64 μg/ml).

**Conclusions:**

Our results emphasize the necessity of periodic testing for antibiotic susceptibility in this geographic region. Based on our *in vitro* examinations, fluoroquinolones could be the most effective drugs for the therapy of *M. bovis* infections in Hungary. However, current antimicrobial use policies have to be taken into account to avoid further antibiotic resistance development and to reserve fluoroquinolones for the treatment of severe infections which have responded poorly to other classes of antimicrobials.

## Background

*Mycoplasma bovis* is a widely distributed pathogen, first isolated in the USA in 1961 from a case of severe mastitis in cattle [1]. It is associated with various diseases in cattle including calf pneumonia, mastitis, arthritis, otitis media and genital disorders [2]. *M. bovis* is considered responsible for a quarter to a third of economic losses in the cattle industry caused by respiratory diseases [3].

In Hungary, the average seropositivity rate of individual animals was found to be 11.3%, in certain herds it even exceeded 50.0%. Tested by enzyme-linked immunosorbent assay the overall rate of seropositive herds was 64.7% [4]. With the exception of seroprevalence on individual level, these values are relatively high in a European context [3,5].

Since no effective vaccine is available against *M. bovis*, adequate housing and appropriate antibiotic treatment are promoted in the control of the diseases caused by this agent. Antibiotic therapy of mastitis has often failed, but antimicrobial treatment of pneumonia has shown some success and it may help reduce economic losses [3,6]. Mycoplasmas are intrinsically resistant to β-lactam antimicrobials and sulphonamides, because they do not possess a cell wall and do not synthesize folic acid. Mycoplasmas are generally susceptible to antibiotics that affect protein (tetracyclines, macrolides, lincosamides, phenicols) or nucleic acid synthesis (fluoroquinolones) [2]. The decreased effectiveness of certain antimicrobial agents (spectinomycin, oxytetracycline and tilmicosin) traditionally used in the therapy of mycoplasma infections was reported in Europe [7].

The aim of this study was to determine the susceptibility of 35 Hungarian *M. bovis* isolates to eleven antibiotics using the microbroth dilution method.

## Methods

**Figure 1:**
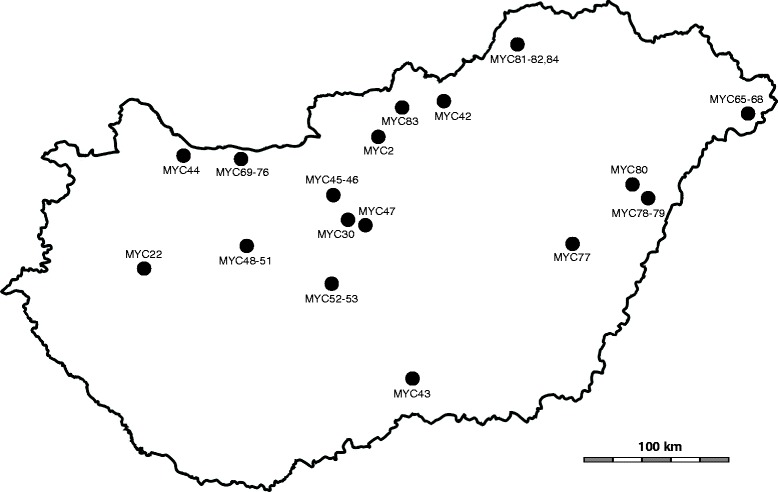
**Map of Hungary showing the geographical origins of the 35** ***M. bovis*** **isolates tested.**

Thirty-five *M. bovis* strains originating from dairy herds located in different parts of Hungary were tested in this study (Table 1, Figure 1). The samples were collected during routine diagnostic examinations or necropsies between 2010 and 2013. Ethical approval was not required for the study as all samples were collected during routine diagnostic examinations or necropsies. Nasal swabs, lung samples and a single lymph node were homogenized in 2 ml of *Mycoplasma* broth medium (pH 7.8) (Thermo Fisher Scientific Inc./Oxoid Inc./, Waltham, MA) supplemented with 0.5% (w/v) sodium pyruvate, 0.5% (w/v) glucose and 0.005% (w/v) phenol red and cultured at 37°C in a 5% CO_2_ atmosphere. Following colour change (red to yellow shift) the cultures were inoculated onto solid *Mycoplasma* media (Thermo Fisher Scientific Inc. /Oxoid Inc./) and were incubated at 37°C and 5% CO_2_ for 3 days, until visible colonies appeared. Mixed cultures were filter cloned only once to exclude contaminant *Mycoplasma* species and to minimize *in vitro* mutations of the isolates. DNA extraction was performed using the QIAamp DNA Mini Kit (Qiagen Inc., Hilden, Germany) according to the manufacturers’ instructions for Gram-negative bacteria. All isolates were identified by polymerase chain reaction (PCR) targeting the *uvr*C gene of *M. bovis* [8]. The purity of the cultures (e.g. to exclude *M. arginini* or other *Mycoplasma* spp. contamination) was confirmed by a universal *Mycoplasma* PCR system targeting the 16S/23S rRNA intergenic spacer region in Mollicutes [9] followed by sequencing on an ABI Prism 3100 automated DNA sequencer (Applied Biosystems, Foster City, CA), sequence analysis and BLAST search. The same once filter cloned passage of each *M. bovis* strain was submitted for a 4 gene based multi-locus sequence typing (MLST) and the sequencing data confirmed the purity of the isolates at strain level (i.e. not more than one *M. bovis* strain in the culture) [10]. Mixed primary cultures which failed to be purified by a single filter cloning were excluded from the study (data not shown). Aliquots of the third passage of purified cultures were stored frozen at −70°C until required. The number of colour changing units (CCU) was calculated by microplate dilution method, from the lowest dilution showing colour change after one week of incubation [11,12].

**Table 1:** **Background information and MIC data of the 35 Hungarian** ***M. bovis*** **isolates included in this study**

**Sample ID**	**Origin of herd**	**Date**	**Sample source**	**MIC values (μg/ml)**										
				**Fluoroquinolones**			**Aminoglycosides**		**Tetracyclines**		**Macrolides**		**Phenicol**	**Lincosamide**
				**Danofloxacin**	**Enrofloxacin**	**Marbofloxacin**	**Gentamicin**	**Spectinomycin**	**Oxytetracycline**	**Tetracycline**	**Tilmicosin**	**Tylosn**	**Florfenicol**	**Lincomycin**
PG45	Connecticut	1961	Lung	0.156	0.156	0.625	4	4	2	0.25	0.5	0.5	4	1
MYC2	Püspökhatvan	2011	Lung	0.156	0.156	0.625	2	2	16	4	128	16	4	1
MYC22	Sümeg	2012	Lung	0.156	0.312	0.625	4	256	64	16	128	128	8	64
MYC30	Bugyi	2012	Lung	0.156	0.156	0.625	4	256	32	8	128	128	4	64
MYC42	Nemti	2012	Lung	0.156	0.156	0.625	8	4	64	8	128	32	8	1
MYC43	Zsana	2012	Lung	0.156	0.156	0.312	4	256	64	16	128	128	8	64
MYC44	Győrszentiván	2012	Lung	10	10	10	2	256	64	8	128	128	8	64
MYC45	Budapest	2012	Lung	10	10	10	2	256	64	8	128	128	4	64
MYC46	Budapest	2012	Lung	10	10	10	4	256	64	8	128	128	8	64
MYC47	Dabas	2012	Lung	0.156	0.156	0.625	8	256	64	8	128	128	8	64
MYC48	Ősi	2012	Nasal swab	0.156	0.156	0.625	8	256	64	16	128	128	4	64
MYC49	Ősi	2012	Nasal swab	0.156	0.156	0.625	8	256	64	16	128	128	4	64
MYC50	Ősi	2012	Lung	0.156	0.156	0.625	4	256	64	8	128	128	4	64
MYC51	Ősi	2012	Nasal swab	0.156	0.08	0.312	4	256	64	8	128	128	4	64
MYC52	Solt	2012	Lung	0.156	0.156	0.312	8	4	2	0.25	0.5	0.5	4	0.5
MYC53	Solt	2012	Lung	0.156	0.156	0.625	16	4	2	0.25	0.5	0.5	4	1
MYC65	Csengersima	2012	Nasal swab	0.156	0.156	0.625	2	2	64	16	128	8	4	0.5
MYC66	Csengersima	2012	Nasal swab	0.156	0.156	0.625	8	4	64	8	128	16	8	1
MYC67	Csengersima	2012	Lung	0.08	0.08	0.312	4	4	16	4	128	16	8	2
MYC68	Csengersima	2012	Lung	0.156	0.156	0.625	4	4	32	4	128	16	4	0.5
MYC69	Komárom	2013	Nasal swab	0.156	0.156	0.625	2	4	32	8	128	32	8	1
MYC70	Komárom	2013	Nasal swab	0.156	0.156	0.625	4	2	32	4	128	32	4	1
MYC71	Komárom	2013	Nasal swab	0.156	0.156	0.625	4	2	32	4	128	32	4	1
MYC72	Komárom	2013	Nasal swab	0.156	0.156	0.625	4	4	32	4	128	32	4	1
MYC73	Komárom	2013	Nasal swab	0.156	0.156	0.625	4	4	32	8	128	32	4	1
MYC74	Komárom	2013	Nasal swab	0.156	0.156	0.625	4	4	32	8	128	16	4	1
MYC75	Komárom	2013	Nasal swab	0.156	0.08	0.312	2	2	32	4	128	32	4	1
MYC76	Komárom	2013	Nasal swab	0.156	0.156	0.625	4	4	64	8	128	16	8	2
MYC77	Kertészsziget	2010	Lung	0.312	0.156	0.625	2	256	64	8	128	128	4	64
MYC78	Hosszúpályi	2011	Lung	0.156	0.156	0.625	4	256	64	8	128	128	4	64
MYC79	Hosszúpályi	2011	Lung	0.156	0.156	0.625	8	256	64	16	128	128	8	64
MYC80	Ebes	2011	Lymph node	0.156	0.156	0.625	4	256	32	4	128	128	4	64
MYC81	Felsőnyárád	2013	Lung	0.156	0.156	0.625	8	256	64	8	128	128	4	64
MYC82	Felsőnyárád	2013	Nasal swab	0.156	0.156	0.625	4	256	64	8	128	128	8	64
MYC83	Alsótold	2013	Lung	0.312	0.156	0.625	4	256	64	8	128	128	8	64
MYC84	Felsőnyárád	2013	Nasal swab	0.156	0.156	0.625	4	256	64	8	128	128	4	64

The following antimicrobial agents were examined during the microbroth dilution tests: three fluoroquinolones: danofloxacin (batch SZBA019XV), enrofloxacin (batch SZBA336XV) and marbofloxacin (batch SZBC248XV); two aminoglycosides: gentamicin (batch 051K17475V) and spectinomycin (batch SZBB166XV); two tetracyclines: oxytetracycline (batch SZBC320XV) and tetracycline (batch SZBA140XV); two macrolides: tilmicosin (batch SZBC345XV) and tylosin (batch SZBB160XV); one phenicol: florfenicol (batch SZBC223XV) and one lincosamide: lincomycin (batch SZBC340XV); all products originated from VETRANAL, Sigma-Aldrich, Germany. They were diluted and stored according to the recommendations of Hannan [11]. Stock solutions of 1 mg/ml enrofloxacin, danofloxacin and marbofloxacin were prepared in 0.1 M NaOH; stock solution of 1 mg/ml florfenicol was prepared in 96% ethanol and in sterile distilled water; and the rest of the stock solutions of 1 mg/ml were prepared in sterile distilled water. All aliquots were stored at −70°C until needed, and dilutions were freshly prepared for each microtest. Twofold dilutions were prepared in the range 0.039-10 μg/ml for fluoroquinolones, 0.125-32 μg/ml for florfenicol, 0.25-64 μg/ml for gentamicin, tetracyclines and lincomycin, 0.5-128 μg/ml for macrolides and 1–256 μg/ml for spectinomycin.

The microbroth dilution test was performed as recommended by Hannan [11] using 10^4^-10^5^ CCU/ml of each strain. In brief, the 96-wells microtiter plates were designed to contain growth control (broth medium without antibiotic), sterility control (broth medium without antibiotic and *Mycoplasma* inoculum) and pH control (broth medium adjusted to pH 6.8) wells. *Mycoplasma* broth medium (pH 7.8) (Thermo Fisher Scientific Inc./Oxoid Inc./) supplemented with 0.5% (w/v) sodium pyruvate, 0.5% (w/v) glucose and 0.005% (w/v) phenol red was used as a culture medium. The duplicates of three clinical isolates and the duplicate of the type strain (*M. bovis* PG45, NCTC 10131) were tested on each 96-well microtiter plates.

The MIC (minimal inhibitory concentration) value of each isolate was defined as the lowest concentration of the antibiotic that completely inhibits the growth in the broth (no pH and colour change) after a one week incubation period [12]. MIC_50_ and MIC_90_ values were defined as the lowest concentrations that inhibit 50% and 90% of bacterial isolates. The type strain (*M. bovis* PG45, NCTC 10131) was used for the quality control of MIC determination (Table 1).

## Results

Our MIC values of *M. bovis* type strain PG45 were identical with values previously obtained for danofloxacin, enrofloxacin, marbofloxacin, spectinomycin, tilmicosin and tylosin using the microbroth dilution method Table 1; [12,13]. The MIC value of PG45 (2 μg/ml) for oxytetracycline was within the range of previously published studies applying microbroth dilution test (0.1/0.125/0.16/4 μg/ml) [6,11-13]. The MIC value (1 μg/ml) of PG45 for lincomycin was higher than in a previous study (0.25 μg/ml) [13]. For gentamicin, tetracycline, and florfenicol data determined by microbroth dilution test were not available. Our results for type strain PG45 were consistent throughout the study.

**Figure 2:**
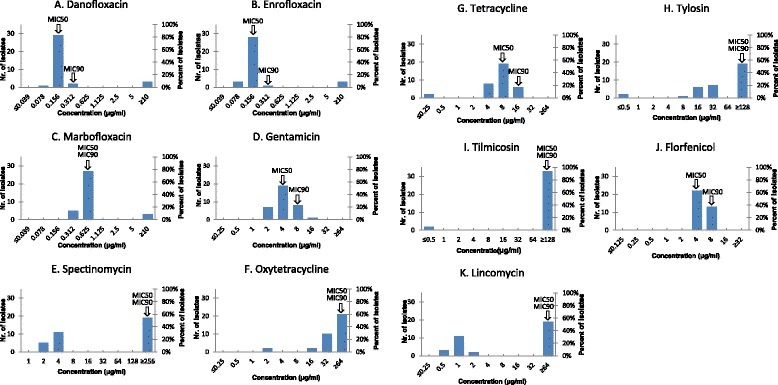
**MIC distribution of the Hungarian isolates for each antibiotic tested in this study.** Arrows indicate the MIC50 and MIC90 values.

The MIC values of the eleven antimicrobial agents obtained from the examinations of the Hungarian *M. bovis* isolates are shown in Figure 2 and listed in Tables 1 and 2. Strains with elevated MIC values were found in the case of all applied antibiotics. Fluoroquinolones were found to be the most active compounds *in vitro*. The antibiotic susceptibility profiles of the Hungarian strains were consistent within the tested group of fluoroquinolones (Figure 2A-C). Three isolates (MYC44, MYC45 and MYC46) had high MIC values (≥10 μg/ml) to danofloxacin, enrofloxacin and marbofloxacin, while the rest of the strains were inhibited by these antimicrobial agents with MICs ≤0.312 or 0.625 μg/ml. The MICs for gentamicin clustered steadily around the MIC_50_ value (4 μg/ml) (Figure 2D). MIC values of spectinomycin divided the strains into two distinct populations, with 48% of isolates yielding MICs of ≤4 μg/ml and the rest clustering with MICs ≥256 μg/ml (Figure 2E). Two *M. bovis* isolates (MYC52 and MYC53) originating from the same herd were inhibited by both tetracyclines and macrolides with low MIC values (Figure 2F-I). Among the macrolides, the MICs of tilmicosin showed bimodal distribution, as two strains yielded MICs ≤0.5 μg/ml, while the rest yielded MICs ≥128 μg/ml. The narrow range of MIC values (4–8 μg/ml) of florfenicol is demonstrated on Figure 2J. MICs for lincomycin also clustered the strains into a group with MICs ≤2 μg/ml and with MICs ≥64 μg/ml (Figure 2K).

**Table 2:** **Summary of range, mode, MIC**_**50**_ **and MIC**_**90**_ **values of the 35** ***M. bovis*** **strains isolated from cattle in Hungary**

		**Range**	**Mode**	**MIC**_**50**_	**MIC**_**90**_
Fluoroquinolones	Danofloxacin	0.078 to ≥10	0.156	0.156	0.312
	Enrofloxacin	0.078 to ≥10	0.156	0.156	0.312
	Marbofloxacin	0.312 to ≥10	0.625	0.625	0.625
Aminoglycosides	Gentamicin	2 to 16	4	4	8
	Spectinomycin	2 to ≥256	≥256	≥256	≥256
Tetracyclines	Oxytetracycline	2 to ≥64	≥64	≥64	≥64
	Tetracycline	≥0.25 to 16	8	8	16
Macrolides	Tylosin	≥0.5 to ≥128	≥128	≥128	≥128
	Tilmicosin	≥0.5 to ≥128	≥128	≥128	≥128
Phenicol	Florfenicol	4 to 8	4	4	8
Lincosamide	Lincomycin	0.5 to ≥64	≥64	≥64	≥64

All values are expressed as μg/ml.

Isolates originating from the same herd showed similar antibiotic susceptibility profiles (Table 1).

## Discussion

Gerchman et al. [13] studied 11 *M. bovis* strains isolated from cattle imported from Hungary to Israel between 2005 and 2007. The most active compounds found during *in vitro* examinations were fluoroquinolones (danofloxacin, enrofloxacin and marbofloxacin), which is in accordance with our results, except that the MIC values described before were higher than the ones detected in this study (MIC_90_ 1.25 μg/ml, 1.25 μg/ml, 5 μg/ml versus 0.312 μg/ml, 0.312 μg/ml, 0.625 μg/ml). Decreased spectinomycin susceptibility was detected in the strains from the imported animals (MIC_90_ > 1024 μg/ml obtained with E-test method), which is consistent with our results (MIC_90_ ≥ 256 μg/ml). In contrast to the results obtained by Gerchman et al. [13] (4 μg/ml, 8 μg/ml, 1 μg/ml) the MIC_90_ values of oxytetracycline (≥64 μg/ml), tilmicosin (≥128 μg/ml) and tylosin (≥128 μg/ml) yielded in the present study indicate limited susceptibility to these antibiotics. The comparison of the results of the previous publication and the present study emphasize the importance of the systematic monitoring of antibiotic susceptibility of *M. bovis* strains in the region [13,14].

Fluoroquinolones inhibited the growth of the majority of the Hungarian *M. bovis* strains at low MIC values (with only 3 exceptions), confirming previous observations that this group of antimicrobial agents is effective against *M. bovis* [6,7,13-18]. MIC values of marbofloxacin were remarkably higher than that of danofloxacin and enrofloxacin. The observed difference, first noted by Gerchman et al. [13] is probably due to the increased use of marbofloxacin during the past years [13]. Extremely high MIC values for fluoroquinolones (≥10 μg/ml) were found in strains MYC44-46. The similarity in the resistance profile of these three strains is consistent with the results of a previous genetic study in the country, where these strains clustered into a separate subclade by MLST [10].

Most Hungarian *M. bovis* strains included in the present examination showed moderate susceptibility to gentamicin, with similar or lower MIC values (MIC_90_ 8 μg/ml) than isolates from Belgium and Israel (MIC_90_ 6 μg/ml, 32 μg/ml) [13,19]. Spectinomycin, another member of the aminoglycosides, was used traditionally as an active compound against *M. bovis* and it is still considered effective in Japan [6,12,14]. However, high MIC values of spectinomycin (≥256 μg/ml) were observed in more than half of the studied Hungarian isolates, which is in agreement with recent reports from other countries [7,13,15-17,19], confirming a globally emerging resistance to spectinomycin.

Heterogenic profiles of *M. bovis* susceptibility to tetracyclines are reported from all over the world [6,7,13,14,16,19]. Only two Hungarian isolates showed low MIC value to oxytetracycline and tetracycline, demonstrating the high level of resistance to tetracyclines among the strains. In accordance with our results, increasing resistance to oxytetracycline was reported previously in Britain, Belgium, Japan and France [7,14,16,19].

All but two of the Hungarian *M. bovis* strains showed high level of resistance to macrolides, with MIC_90_ values (≥128 μg/ml) consistent with previously published data, suggesting that macrolides are losing their efficacy on mycoplasmas [6,7,13,14,16]. For example an earlier clinical study demonstrated the effective use of tilmicosin [20] but another study [21] twelve years later already demonstrated the ineffectiveness of tilmicosin against *M. bovis in vivo* which also emphasizes the spread of antibiotic resistance due to the escalating use of antibiotics in veterinary practice. In the present study MICs of tilmicosin grouped around two distinct values, while the distribution of MICs of tylosin was gradually dispersed (Figure 2H-I). MIC values of tylosin were lower (8-128 ≤ μg/ml) or similar to MICs of tilmicosin (≥128 μg/ml). Similar observations were reported in the case of *M. bovis* strains by Gerchman et al. [13] and in the case of *M. gallisepticum* isolates by Jordan and Horrocks [22]. The slower development of tylosin resistance is supposed to be the cause of the difference between the MIC values of these antibiotics [23], and our results provide further evidence for this phenomenon.

Outstandingly low MIC values of all tetracyclines and macrolides were observed in two Hungarian isolates originating from the same herd (MYC52-53) and in the case of the reference PG45 strain. These three strains were closely related and they also formed a separate genetic clade in the MLST analysis performed previously [10].

The Hungarian isolates showed high MIC values to florfenicol. The MIC_90_ values (8 μg/ml) were similar to values obtained earlier in the United Kingdom (16 μg/ml), USA (4 μg/ml) and France (16 μg/ml) [6,7,16].

MIC_90_ values of lincomycin (≥64 μg/ml) were higher than the ones (1 μg/ml, 8 μg/ml, 64 μg/ml) described elsewhere [12,14,19]; and more than half of the strains isolated from cattle in Hungary demonstrated high MIC values to this member of lincosamides.

The results of *in vitro* antibiotic susceptibility tests can only predict the expected *in vivo* efficacy of the antibiotics, thus they only indicate the potential usefulness of a certain antimicrobial agent in the therapy. Standard breakpoints (susceptible, intermedier, resistant categories) have not yet been defined for the interpretation of *M. bovis* susceptibility to antibiotics [24], but several authors derived breakpoints for mycoplasmas from breakpoints of other bovine pathogens, and in some cases values were adopted from other host species [6,13-17]. Taking into account all these criteria, fluoroquinolones seem to be the most active compounds *in vivo* against the *M. bovis* strains existing in Hungary. Although the *in vitro* antibiotic susceptibility tests are promising, the use of fluoroquinolones against *M. bovis* could be controversial *in vivo*. In the United Kingdom Nicholas and Ayling [3] reported on a study where the monthly fluoroquinolone treatment repeated over three months did not prevent the development of respiratory disease caused by *M. bovis*.

## Conclusions

The present study determined the antibiotic susceptibility profiles of 35 *M. bovis* strains isolated from cattle in Hungary and it highlighted the importance of regular testing of antibiotic susceptibility in the region. Our results confirmed the increasing resistance to antibiotics commonly used for the treatment of mycoplasma infections, primarily to tetracyclines and macrolides. Based on the presented *in vitro* examinations, fluoroquinolones could be the most effective in the therapy of *M. bovis* infections in Hungary. However, the identification of three fluoroquinolon resistant isolates lends support for the EU recommendation that prudent antimicrobial use policies have to be strictly observed when members of this antibiotic group are applied [25]. In order to avoid the development of resistance fluoroquinolones should only be used based on the results of susceptibility testing and in cases of severe infections when treatment failed with other classes of antimicrobials.

## Abbreviations

MIC: Minimal inhibitory concentration
MLST: Multi-locus sequence typing
